# Altered levels of memory T cell subsets and common γc cytokines in *Strongyloides stercoralis* infection and partial reversal following anthelmintic treatment

**DOI:** 10.1371/journal.pntd.0006481

**Published:** 2018-05-24

**Authors:** Anuradha Rajamanickam, Saravanan Munisankar, Yukti Bhootra, Chandra Kumar Dolla, Kannan Thiruvengadam, Thomas B. Nutman, Subash Babu

**Affiliations:** 1 National Institute of Health-NIRT-International Center for Excellence in Research, Chennai, India; 2 National Institute for Research in Tuberculosis, Chennai, India; 3 Laboratory of Parasitic Diseases, National Institute of Allergy and Infectious Diseases, National Institutes of Health, Bethesda, Maryland, United States of America; University Hospital of Bonn, GERMANY

## Abstract

**Background:**

CD4^+^ and CD8^+^ T cells are central players in immunity to helminth infections. However, the role of T cell subsets in human helminth infections is not well understood. In addition, the common γc cytokines, IL-2, IL-4, IL-7, IL-9 and IL-15 play an important role in the maintenance of these CD4^+^ and CD8^+^ T cell subsets.

**Methods:**

To examine the major T cell subsets and their association with the common γc cytokines, the absolute numbers of CD4^+^ and CD8^+^ naïve, central memory, effector memory and effector cells and the plasma levels of IL-2, IL-4, IL-7, IL-9 and IL-15 were measured in *Strongyloides stercoralis* (*Ss*) infected (INF, n = 60), helminth—uninfected (UN, n = 58) and in post treatment INF individuals.

**Results:**

*Ss* infection is characterized by significantly increased absolute numbers of naïve and decreased absolute numbers of central and effector memory CD4^+^ T cells in comparison to UN individuals. No significant difference in the numbers of CD8^+^ T cell subsets was observed between the groups. The numbers of naïve cells and central memory CD4^+^ T cells were significantly reversed after anthelmintic treatment. Circulating levels of IL-2, IL-7 and IL-15 were significantly diminished, whereas the levels of IL-4 and IL-9 were significantly increased in INF compared to UN individuals. Following anthelminthic treatment, IL-2, IL-7 and IL-15 levels were significantly increased, while IL-4 and IL-9 levels were significantly decreased. Our data also showed a significant positive correlation between the levels of IL-7 and the numbers of central and effector memory CD4^+^ T cells.

**Conclusion:**

*Ss* infection is characterized by alterations in the absolute numbers of CD4^+^ T cell subsets and altered levels of common γc cytokines IL-2, IL-4, IL-7, IL-9 and IL-15; alterations which are partially reversed after anthelmintic treatment.

## Author summary

*Strongyloides stercoralis* (*Ss*), an intestinal nematode, the causative agent of strongyloidiasis, infects 30–100 million people worldwide. *Ss* infection is often clinically asymptomatic and long lasting due, in large part, to the parasites auto-infective life cycle and their ability to modulate the host immune system. Th1 cells are down modulated and Th2 cells are essential for fighting against helminth infections. T cells proliferate in response to common γc dependent cytokine signaling. The role of CD4^+^ and CD8^+^ T cell subset distribution and the association between memory T cell subsets and the common γc cytokines (IL-2, IL-4, IL-7, IL-9 and IL-15) in helminth infections has not been explored well. We examined the *ex-vivo* phenotypic profile of CD4^+^ and CD8^+^ T cell subsets and the circulating levels of common γc cytokines in *Ss*-infected, uninfected and post treatment individuals. *Ss* infected individuals showed alterations in the T cell subset distribution and these alterations were partially reversed following anthelminthic treatment. This was associated with altered plasma levels of IL-2, IL-4, IL-7, IL-9 and IL-15 and partial reversal following anthelminthic treatment. IL-7 exhibited significant positive association with central and effector memory CD4^+^ T cells. Our study would provide stimulus to examine further about the function of T cell subset distribution and the role and association of common γc cytokines with parasitic infections.

## Introduction

*Strongyloides stercoralis (Ss)*, a soil transmitted nematode that resides in the small intestine of humans, infects approximately 30–100 million people worldwide [1]. The clinical manifestations of *Ss* infection can range from the clinically asymptomatic to, at its most severe, the potentially fatal hyperinfection syndrome. *Ss* infection is associated with down modulation of Th1 and Th17 responses and up-regulation of Th2 and Th9 CD4^+^ T cell responses [2, 3]. How *Ss* infection influences CD8^+^ T cell responses has not been studied in detail. In addition, very little is known about CD4^+^ or CD8^+^ memory T cell subset distribution in *Ss* infection.

Common cytokine receptor γ-chain family (γc cytokines) are associated with the process of memory T cell generation [4–6]. The sharing of the γ chain by their receptors, common downstream signalling pathways, link members of this cytokine family functionally. Data reveal that IL-2, IL-4, IL-7, IL-9 and IL-15 participate in the initiation of T cell responses and that some of these cytokines are vital for the development or maintenance of memory T cells [7]. Murine studies have shown that different cell types produce the major γc cytokines IL-7 and IL-15, that play important roles in the maintenance of CD4^+^ [8] and CD8^+^ T cells [9, 10]. Human studies also have shown that T cells proliferate in response to common γc dependent cytokine signaling [11, 12], but the association between memory T cell subsets and these common γc cytokines in helminth infections has not been examined. The common γc cytokines, IL-2, IL-7 and IL-15 play an important role in peripheral T cell growth and survival [4–6]. However, the effects of helminth infection on common γc cytokine—IL-2, IL-4, IL-7, IL-9 and IL-15- levels have not been explored in *Ss* infection.

We hypothesized that *Ss* infection would be associated with alterations in memory T cell subset distribution, alterations that could be reflective of changes in IL-2, IL-4, IL-7, IL-9 and IL-15. We, therefore, examined the ex vivo phenotypic profile of CD4^+^ and CD8^+^ memory T cell subsets and the circulating levels of common γc cytokines (IL-2, IL-4, IL-7, IL-9 and IL-15) in *Ss*-infected (INF) and -uninfected (UN) individuals. We also examined the effect of anthelmintic treatment on the distribution of these memory T cell subsets and the cytokines.

## Material and methods

### Ethics statement

All individuals (age between 18–65 years) were examined as part of a natural history study protocol approved by Institutional Review Boards of the National Institute of Allergy and Infectious Diseases (USA) and the National Institute for Research in Tuberculosis (India), and informed written consent was obtained from all participants.

### Study population

We studied 118 individuals comprised of 60 clinically asymptomatic, *Ss*-infected (hereafter INF) individuals and 58 *Ss*-uninfected, endemic healthy (hereafter UN) individuals in Sirukalathur village, Kanchipuram District, Tamil Nadu, South India (Table 1). These individuals were all recruited from a rural population by screening of individuals for helminth infection by stool microscopy and serology as described previously [13–15]. None had previous anthelmintic treatment or a history of prior helminth infection. Follow up among the INF individuals was performed at 6 months following treatment. These individuals were different from our previous studies on serum cytokines in *Ss* individuals [16].

**Table 1:** Baseline demographics and hematology of study population.

Study Demographics	INF	UN	
**Number**	n = 60	n = 58	
**Gender (Male/Female)**	37/23	38/20	
**Median age (range)**	36 (20–61)	40 (20–60)	
**NIE ELISA**	Positive	Negative	
**Stool microscopy**	Positive for *Ss* and negative for other intestinal helminths	Negative for all intestinal helminths	
**Haematology profile**			**p value**
**Red blood cell count,x10**^**6**^**/ul**	4.5 (3.5–6.06)	4.07 (2.11–5.84)	p = 0.0391
**White blood cell count, x10**^**3**^**cells/ul**	9400 (5800–16900)	8700 (5300–15400)	NS
**Lymphocyte count, cells/ml**	2515 (1553–3711)	2463 (1486–4411)	NS
**Neutrophil count, cells/ml**	4803 (3111–11069)	4997 (2562–9933)	NS
**Monocyte count, cells/ml**	662 (378–1000)	635 (335–1100)	NS
**Eosinophil count, cells/ml**	800 (102–3519)	343 (73–9933)	p<0.0001
**Basophil count, cells/ml**	88 (12–306)	85 (31–387)	NS

The values represent geometric mean and range

*Ss* infection was diagnosed by the presence of IgG antibodies to the recombinant NIE antigen as described previously [14, 15]. This was further confirmed by stool microscopy. A single stool sample was obtained and examined for intestinal helminth infection by Kato-Katz technique. Stool samples found to be negative for other intestinal helminths by stool microscopy and positive for *Ss* infection by serology were then subjected to specialized stool examination with nutrient agar plate cultures. Only the individuals who were positive for *Ss* infection by both serology and nutrient agar culture plate technique were selected for this study. Filarial infection was excluded in all study participants by virtue of being negative in tests for circulating filarial antigen. All INF individuals were treated with single dose of ivermectin (12mg) and albendazole (400 mg) and follow—up blood draws were obtained six months later. Follow up examination was done by stool microscopy of a single stool sample using Kato-Katz and nutrient agar plate cultures, which was negative for *Ss* as well as other intestinal helminth infection. In addition, serology showed a significant decrease in the IgG titers to NIE antigen.

### Ex vivo analysis

Leukocyte counts and differentials were performed on all individuals using an AcT5 Diff hematology analyzer (Beckman Coulter). All antibodies used in the study were from BD Biosciences (San Jose, CA), BD Pharmingen (San Diego, CA), eBioscience (San Diego, CA), or R&D Systems (Minneapolis, MN). Whole blood was used for ex vivo phenotyping and it was performed on all 118 individuals. Briefly, 250ul aliquot of whole blood was added to a cocktail of monoclonal antibodies specific for various immune cell types. T cell phenotyping was performed using antibodies directed against CD45-Peridinin chlorophyll protein (PerCP; clone 2D1, BD), CD3-AmCyan (clone SK7; BD), CD4-phycoerythrin (PE) Cy7 (clone SK3; BD), CD8-allophycocyanin (APC) H7 (clone SK1; BD), CD45RA-Pacific Blue (clone H1100; Biolegend, Cambridge, UK), and CCR7-FITC (clone 3D12; eBioscience) (S1 Table). Naive cells were classified as CD45RA^+^ CCR7^+^, central memory cells as CD45RA^-^ CCR7^+^, effector memory cells as CD45RA^-^CCR7^-^ and effector cells as CD45RA^+^ CCR7^-^ (S2 Table). Following 30 min of incubation at room temperature, erythrocytes were lysed using 2 ml of FACS lysing solution (BD Biosciences Pharmingen), cells were washed twice with 2 ml of PBS and suspended in 200 ul of PBS (Lonza, Walkersville, MD). Eight- color flow cytometry was performed on a FACS Canto II flow cytometer with FACSDIVA software, version 6 (Becton Dickinson). The gating was set by forward and side scatter, and 1,00000 gated events were acquired. Gating strategy for memory T cell subsets were shown in S1 Fig. Data were collected and analyzed using FLOW JO software (TreeStar, Ashland, OR). Leukocytes were gated using CD45 expression versus side scatter. Total lymphocyte counts were obtained from the hematology profile and the percentage of gated lymphocytes by flow cytometry was used to calculate the absolute numbers of T cell subsets.

### ELISA

Circulating levels of IL-2, IL-4, IL-7 and IL-15 were measured using the Quantikine ELISA kit (R&D Systems) and IL-9 (eBiosciences) were measured by enzyme-linked immunosorbent assay (ELISA), according to the manufacturer’s instructions. The lowest detection limits were as follows: IL-2, 31.2 pg/mL; IL-4, 31.2 pg/mL; IL-7, 7.813 pg/mL; IL-15, 16.625 pg/mL; IL-9, 3.1 pg/mL.

### Statistical analysis

Data analyses were performed using GraphPad PRISM (GraphPad Software, Inc., San Diego, CA, USA). Geometric means (GM) were used for measurements of central tendency. Statistically significant differences were analyzed using the nonparametric Mann-Whitney U test used to compare INF versus UN and Wilcoxon signed rank test was used to compare memory T cell panel and common γ-chain cytokines levels before and after treatment. Multiple comparisons were corrected using the Holm’s correction. Correlations were calculated by the Spearman rank correlation test. Analyses were performed using Graph-Pad PRISM Version 6.0 (GraphPad, San Diego, CA) or R. JMP 13 (SAS) software was used to perform Spearman rank correlation matrix. Logistic regression analysis was used to identify factors that influenced by *Ss* infection. P ≤ 0.05 was considered statistically significant. STATA 15.0 (StataCorp, College Station, Texas, USA) was used for Logistic regression analysis.

## Results

### Study population characteristics

The baseline characteristics and demographics of the study population are shown in Table 1. No significant differences in age, gender, socioeconomic status, or geographical location were observed between the two groups. The baseline hematological features of the study population are also shown in Table 1. As can be seen, INF individuals had few differences in any of the hematological parameters measured with the exception of the RBC and absolute eosinophil counts (AECs) that were higher in INF individuals (p<0.0001).

### *Ss* infection is associated with alterations of CD4^+^ naïve, central memory and effector T cells but not CD8^+^ T cells

**Fig 1:**
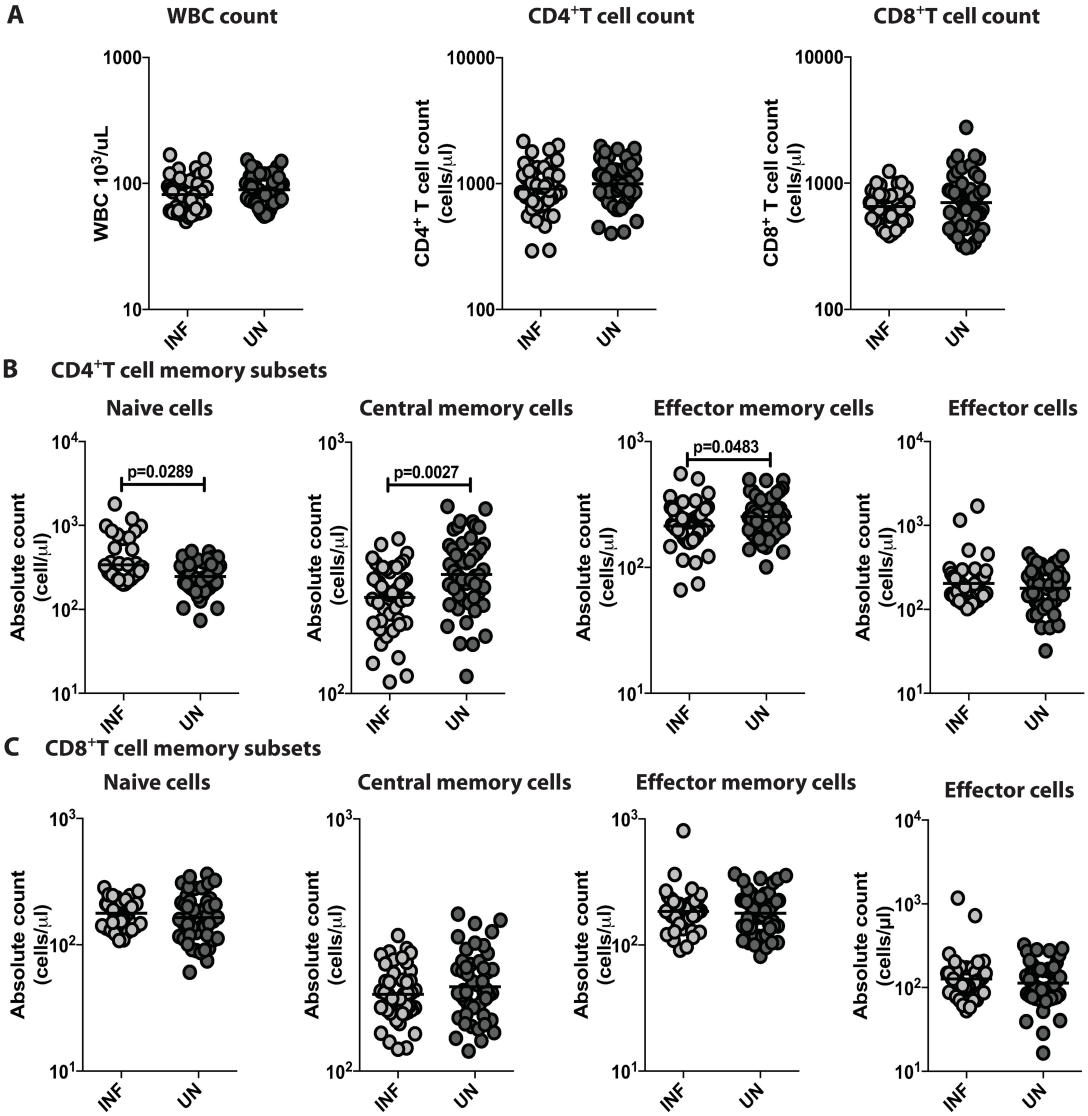
Ss infection is associated with alterations of CD4^+^ naïve, central memory and effector cells but not CD8^+^ T cells. (A) Absolute count of leukocytes, CD4^+^T cells and CD8+T cells in Ss-infected [INF] (n = 60) or un-infected [UN] (n = 58) individuals. (B) Absolute counts of CD4^+^ T cell subsets—naïve cells, central memory, effector memory cells and effector cells. (C) Absolute counts of CD8+T cell subsets—naïve cells, central memory, effector memory cells and effector cells. The data are represented as scatter plots with each circle representing a single individual. P values were calculated using the Mann–Whitney U-test with Holms correction for multiple comparisons.

To determine if *Ss* infection altered absolute numbers of T cells, we measured the absolute leukocyte count and absolute CD4^+^ and CD8^+^ T cell counts in INF and UN individuals. There were no significant differences in absolute leukocyte counts or absolute CD4^+^ and CD8^+^ T cell counts between INF and UN individuals (Fig 1A). To study the distribution of CD4^+^ memory T cell subsets in *Ss* infection, we examined the counts of four different CD4^+^ T cell subsets (naive, central memory, effector memory and effector) in INF and UN individuals at baseline. As shown in Fig 1B, INF had significantly increased numbers of naïve CD4^+^ T cells (GM of 339.1 in INF versus 246.1 in UN (p = 0.0289)) in comparison with UN individuals. In contrast, central memory (GM of 241 in INF versus 297.9 in UN (p = 0.0027)) and effector memory (GM of 213.7 in INF versus 253.2 in UN (p = 0.0483)) CD4^+^ T cell numbers were significantly decreased in INF individuals when compared with UN individuals. There were no significant differences in the counts of naive, central, effector memory, and effector cells in CD8^+^ T cell compartment between INF and UN individuals (Fig 1C). Therefore, *Ss* infection appears to be associated with alterations in the memory subset distribution of CD4^+^ T cells.

### Anthelmintic therapy significantly alters the CD4^+^ memory T cell subset numbers in *Ss* infection

**Fig 2:**
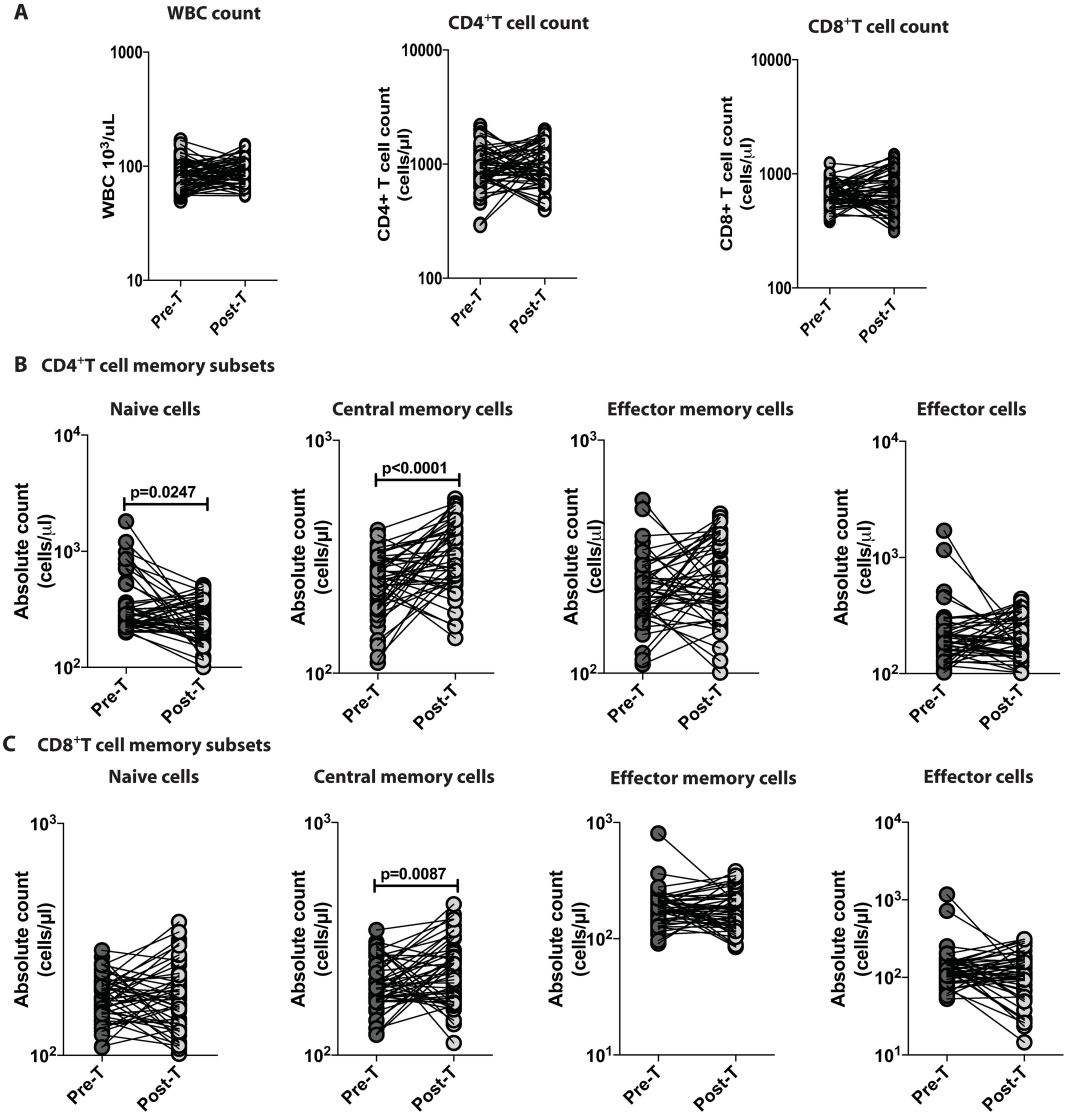
Anthelmintic therapy significantly alters the CD4^+^T cell subset numbers in Ss infection. (A) Absolute count of leukocytes, CD4^+^ T cells and CD8^+^T cells of Ss-infected INF individuals at pre-treatment [pre-Tx] (n = 50) and 6 months following treatment post-treatment [post-Tx] time points. (B) Absolute counts of CD4^+^ T cell subsets—naïve cells, central memory, effector memory cells and effector cells. (C) Absolute counts of CD8^+^T cell subsets—naïve cells, central memory, effector memory cells and effector cells. Data are shown line diagrams with each line representing a single individual. P values were calculated using the Wilcoxon matched pair test with Holms correction for multiple comparisons.

To determine the effect of treatment on absolute leukocyte counts and absolute numbers of CD4^+^ and CD8^+^ T cells, we measured these parameters in INF individuals 6 months following anthelmintic treatment. There were no significant differences in absolute leukocyte counts or absolute CD4^+^ and CD8^+^ T cell counts (Fig 2A). In contrast, as shown in Fig 2B, the absolute numbers of naïve CD4^+^ T cells (GM of 339.1 in pre-treatment (Pre-Tx) compared to 248.6 in post-treatment (Post-Tx) p = 0.0247) were significantly decreased and central memory T cell (GM of 241 in Pre-Tx versus 314.2 in Post-Tx p<0.0001) counts were significantly increased following anthelmintic treatment. As shown in Fig 2C, CD8^+^ T cell central memory counts (GM of 201.8 in Pre-Tx versus 227.5 in Post-Tx, p = 0.0087) were also significantly increased after treatment. Therefore, treatment of Ss infection is associated with partial but significant reversal of the alterations seen in naïve and central memory T cells prior to treatment. Moreover, following treatment, there was an increase in the CD8^+^ central memory T cell compartment.

### *Ss* infection is associated with decreased plasma levels of IL-2, IL-7 and IL-15 and enhanced plasma levels of IL-4 and IL-9

**Fig 3:**
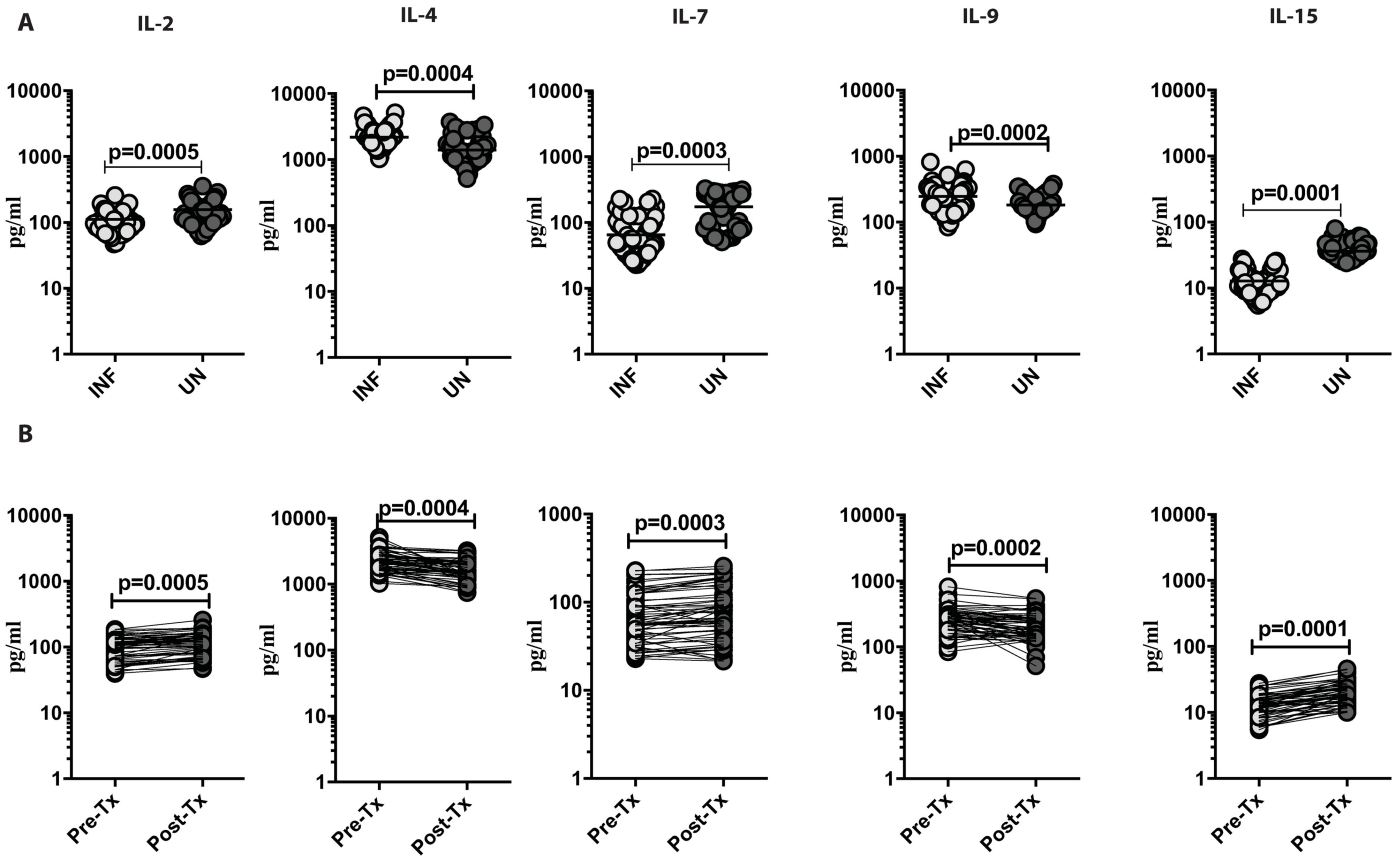
*Ss* infection is associated with decreased plasma levels of IL-2, IL-7 and IL-15 and enhanced plasma levels of IL-4 and IL-9. (A) The plasma levels of common γc cytokines IL-2, IL-4, IL-7, IL-9 and IL-15 were measured in Ss-infected [INF] (n = 60) or un-infected [UN] (n = 58) individuals. The data are represented as scatter plots with each circle representing a single individual. P values were calculated using the Mann–Whitney U-test with Holms correction for multiple comparisons. (B) The plasma levels of common γc cytokines IL-2, IL-4, IL-7, IL-9 and IL-15 were measured in Ss-infected INF individuals at pre-treatment [pre-Tx] (n = 50) and 6 months following treatment post-treatment [post-Tx] time points. The data are represented as line graphs with each line representing a single individual. P values were calculated using the Wilcoxon signed rank test.

Because common γc cytokines play an essential role in homeostasis and expansion of memory T cells, we wanted to understand the association of γc cytokines with *Ss* infection and following treatment. We measured the circulating plasma levels of IL-2, IL-4, IL-7, IL-9 and IL-15 in INF and UN individuals. As shown in Fig 3A, INF had significantly lower serum levels of IL-2 (GM of 111.6 pg/ml in INF versus 156.8 pg/ml in UN, p = 0.0005); IL-7 (GM of 64.72 pg/ml in INF versus 172.5 pg/ml in UN, p = 0.0003) and IL-15 (GM of 12.79 pg/ml in INF versus 36.28 pg/ml in UN, p = 0.0001) in comparison to UN individuals. In contrast, INF individuals had significantly enhanced serum levels of IL-4 (GM of 2196 pg/ml in INF versus 1396 pg/ml in UN, p = 0.0004) and IL-9 (GM of 245.7 pg/ml in INF versus 183.2 pg/ml in UN, p = 0.0002) when compared with UN individuals. Following treatment, IL-2 (fold change 1.183), IL-7 (fold change 1.130), and IL-15 levels (fold change 1.513) levels exhibited an increase from pretreatment levels in INF individuals. In contrast, levels of IL-4 (fold change 0.727) and IL-9 (fold change 0.799) were diminished from pretreatment levels when compared to post treatment levels (Fig 3B) Thus, *Ss* infection is associated with altered levels of common γc cytokine levels and partial reversal following treatment.

### CD4^+^ central memory and effector memory T cell subsets exhibit a positive relationship with IL-7 and but not with other common γc cytokines

**Fig 4:**
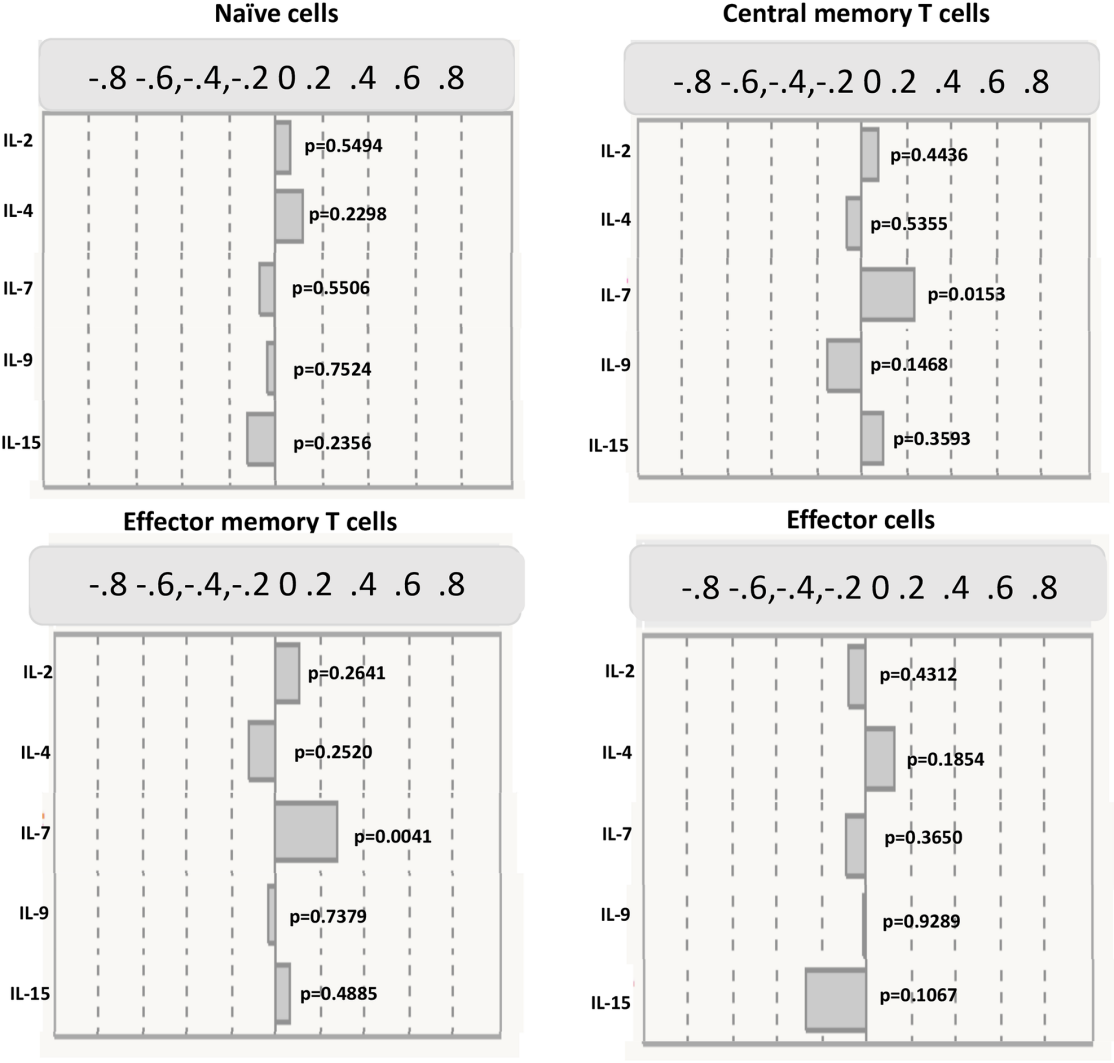
CD4^+^ central memory and effector memory T cell subsets exhibit positive relationship with IL-7 and but not with IL-2, IL-4, IL-9 or IL-15. The correlation between plasma levels of common γc cytokines (IL-2, IL-4, IL-7, IL-9 and IL-15) and CD4^+^ memory T cell subsets (naïve, central memory, effector memory and effector cells) are shown. Fig.4A. shows correlation between CD4^+^ naive T cells and γc cytokines. Fig.4B. illustrates correlation between CD4^+^ central memory T cells and γc cytokines. Fig.4C. depicts correlation between CD4^+^ effector memory T cells and γc cytokines. Fig.4D. describes correlation between CD4^+^ effector cells and γc cytokines in Ss-infected individuals (n = 60). P and r-values were calculated using the Spearman rank correlation test at 95% confidence intervals using JMP software. The upper scale bar represents the r values.

The relationship between the levels of common γc cytokines, IL-2, IL-4, IL-7, IL-9 and IL-15 and CD4+ memory T cell subsets (naïve cells, central memory cells, effector memory cells and effector cells) were next assessed by Spearman correlation. As shown in Fig 4A and 4D, CD4^+^ naïve and effector cells did not reveal any correlation with common γc cytokines (IL-2, IL-4, IL-9 and IL-15). As shown in Fig 4B, the levels of IL-7 exhibited a significant positive correlation with the absolute numbers of CD4^+^ central memory T cells (r = 0.2366; p = 0.0153), whereas other common γc cytokines (IL-2, IL-4, IL-9 and IL-15) did not show any significant correlation with central memory T cell subsets. Similarly, IL-7 (but not other common γc cytokine) levels exhibit a significant positive correlation with CD4^+^ effector memory T cells (r = 0.2413; p = 0.0041) (Fig 4C).

### Logistic regression analysis of CD4^+^ and CD8^+^memory T cell subsets and γc cytokines in *Ss* infection

Logistic regression analysis was done to examine the influence of age, sex or haematological parameters on CD4 and CD8 memory subsets—naïve, central memory, effector memory and effector cells and the γc cytokines IL-2, IL-4, IL-7, IL-9 and IL-15. As shown in Table 2, logistic regression analysis did not reveal any effect of on the CD4^+^ memory subsets between the two groups. Among the γc cytokines, the adjusted odds ratio for IL-15 alone was significant, other cytokines did not show any difference. Similarly, CD8^+^ memory subsets did not show any difference. Thus, *Ss* infected individuals associated with alterations in the T cell subset distribution and altered plasma levels of IL-2, IL-4, IL-7, IL-9 and IL-15.

**Table 2:** Logistic regression analysis for CD4^+^ and CD8^+^memory T cell subsets and γc cytokines.

Factors	OR (95% CI)	p Value	Adjusted OR (95% CI)	p Value
Naïve (CD4^+^)	1.004 (1.001–1.007)	0.012	1.001 (0.994–1.007)	0.951
Central Memory (CD4^+^)	0.992 (0.987–0.997)	0.002	0.978 (0.918–1.041)	0.475
Effector Memory (CD4^+^)	0.956 (0.993–1.001)	0.051	0.998 (0.930–1.070)	0.949
Effector Cells (CD4^+^)	1.002 (0.999–1.004)	0.258	0.996 (0.906–1.095)	0.931
Naïve (CD8^+^)	1.001 (0.995–1.008)	0.743	1.026 (1.002–1.051)	0.036
Central Memory (CD8^+^)	0.995 (0.989–1.002)	0.159	0.993 (0.970–1.016)	0.551
Effector Memory (CD8^+^)	1.001 (0.996–1.005)	0.712	1.007 (0.983–1.032)	0.567
Effector Cells (CD8^+^)	1.002 (0.998–1.005)	0.391	0.980 (0.951–1.011)	0.208
IL2	0.982 (0.974–0.991)	<0.001	0.996 (0.978–1.014)	0.645
IL4	1.002 (1.001–1.003)	<0.001	1.002 (0.999–1.001)	0.806
IL7	0.981 (0.975–0.988)	<0.001	0.993 (0.981–1.004)	0.225
IL9	1.010 (1.005–1.015)	<0.001	1.002 (0.993–1.011)	0.640
IL15	0.456 (0.259–0.802)	0.006	0.865 (0.764–0.980)	0.022

## Discussion

The major subsets of memory CD4^+^ and CD8^+^ T cells can be defined by the expression of CD45RA and CCR7 [17], and these cells can be subdivided into naive, central memory, effector memory and effector T cells in the circulation based on the expression pattern of the above markers. CCR7^+^ memory T cells are termed central memory T cells and are able to home to secondary lymphoid organs and produce high levels of IL-2 but low levels of other cytokines, whereas CCR7^–^ memory T cells are termed effector memory T cells and are able to produce high levels of effector cytokines, exert rapid effector functions, and home to peripheral tissues [18]. CD4^+^ memory T cells have been shown to mediate protection against re-infection in experimental helminth infection [19].

Central memory and effector memory T cells have been shown to play important roles in protective immune responses in animal models of vaccination or protective immunity with central memory T cells dominating the antigen-specific immune response in vaccination experiments [20–22]. Alteration of memory T cell responses may be involved in the modulation of T cell responses in individuals with patent filarial infection, another tissue-invasive helminth parasite [23]. Indeed, effector memory and central memory CD4^+^ T cells are associated with protective immunity in some parasitic infections [24, 25]. In patients with schistosomiasis, proportion of CD4^+^ memory T cells was significantly lower than in uninfected people [26]. Similarly, the present study demonstrated diminished number of effector memory and central memory CD4 cells in *Ss*-infected individuals, with a corresponding increase in naïve CD4 cells. Because central memory T cells have a high proliferative potential required to mediate protection against a number of pathogens [20, 27, 28], the fact that central memory T cells are decreased in *Ss* and other helminth infections (e.g. filarial infections) and fail to proliferate to antigen [29, 30] suggests that central memory T cells could play a role in the immune response to helminth infections in humans.

It is possible that the decreased numbers of effector memory cells in *Ss* infected individuals could be due to increased migration of memory cells from the circulation to mucosal sites. Previous studies on filarial infection have shown that alterations in effector and memory cell population *ex vivo* could contribute to the antigen specific T cell hypo-responsiveness, commonly seen in these infections [23, 31]. In addition, the differences in the T memory cell distributions might be due to alterations in antigen presentation by antigen presenting cells. CD4^+^ effector and memory T cells require more abundant presentation of antigens by antigen presenting cells than do CD8^+^ memory T cells [32]. In the present study, *Ss* infected individuals exhibited increased naïve CD4^+^ T cell counts and decreased central memory and effector memory CD4^+^ T cell counts. The naïve CD4^+^ T cell counts and the central memory CD4^+^ T cell counts were significantly but partially reversed at 6 months following anthelmintic treatment. This is similar to the findings in another helminth infection, wherein schistosome infected individuals showed reversal of memory CD4^+^ T cells after anthelmintic treatment [33]. The increased levels of naïve cells and decreased levels of central memory and effector CD4^+^ T cell numbers could potentially reflect an important component of the chronic immune response to *Ss* infection, especially as the effector compartment also undergoes expansion in size following chemotherapy. Moreover, protective immunity to *S*. *stercoralis* in mice requires CD4^+^ T cells but not CD8^+^ T cells [24]. Hence, it is not surprising to observe changes related to CD4^+^ T cell memory subsets in *Ss* infection.

The common γc cytokines play an essential role in peripheral T cell expansion, function, and survival [34] and are also vital growth factors for T cells [11]. IL-2 is essential for the induction of T_H_2 cell differentiation [35, 36] and is mainly expressed by T cells, primarily the CD4^+^ Th1 subsets and also by stimulated CD8^+^ T cells and dendritic cells (DCs). IL-2 can induce the proliferation and survival of TCR-activated human and mouse T cells [37] and is required for sustained expansion of T cell populations [38]. In our previous study, we have shown that IL-2 levels have been significantly diminished in *Ss* infection when compared to uninfected individuals [16]. Our current study extends and corroborates these findings.

IL-4 is the main cytokine necessary for the induction of T_H_2 cells. IL-4 also has an important role in allergy and immunoglobulin class switching [39]. Protective immunity to *Ss* larvae in mice is dependent on CD4^+^ T cells, and these cells typically produce IL-4 [24]. IL-9 is produced by a subset of activated CD4 T cells [40] and it provokes the activation of epithelial cells, B cells, eosinophils and mast cells [41]. IL-9 plays the role as T cell growth factor during the late phase of an immune response [42]. Animal studies revealed that Th9 cells have been associated in resistance against intestinal helminth infection [43]. We have previously examined the role of Th2 and Th9 cells in *Ss* infection [2, 3]. In this study, we extend these findings and demonstrate that IL-4 and IL-9 levels were significantly enhanced in INF individuals. This confirms our previous data demonstrating elevated IL-4 and IL-9 responses in *Ss* infection and its reversal following anthelmintic therapy [16].

IL-7 is an important cytokine, an essential survival factor for T cells, plays a major role in T cell homeostasis, expansion of memory CD4^+^ T cells and proliferation of naïve and CD8^+^ T cells [10, 44]. Our data showed diminished levels of IL-7 in *Ss* infected individuals when compared to uninfected individuals at baseline. Our data also revealed that IL-7 levels exhibited significant positive correlation with CD4^+^ central memory and effector memory subsets. IL-15 is a pleiotropic cytokine, which has different roles in the innate and adaptive immune system, including the development, activation, homing and survival of immune effector cells and antigen-independent expansion of naive and memory CD4^+^ and CD8^+^ T cells [45, 46]. In the present study, IL-15 levels were also diminished in *Ss* infected individuals in comparison with uninfected individuals. There was a significant reversal of IL-7 and IL-15 levels following anthelmintic treatment. However, IL-15 did not exhibit any relationship with memory CD4^+^ T cell subsets. Thus, our data provides evidence that changes in the plasma levels of the common γc cytokines, IL-2, IL-4, IL-7, IL-9 and IL-15 are associated with the differential memory T cell compartment alterations seen in *Ss* infected individuals. However, only IL-7 appears to exhibit a significant correlation. Our study also clearly depicts the alteration of the cytokine profile with treatment. IL-2, IL-7 and IL-15 could possibly serve as lymphoid growth factors and could underlie novel strategies for immune recovery and the optimization of immune therapies in helminth infections.

Our study has limitations in that we have not explored the functional significance of these changes in cellular subsets. However, it does provide impetus to further examine the function of these T cell subsets in Ss as well as other parasitic infections. Nevertheless, our work highlights the growing importance of these subsets and the role of common γc cytokines to parasitic infections.

## Supporting information

S1 Fig A representative flow cytometry plot from a *Ss* individual showing the gating strategy for naïve, central memory and effector memory cells from CD4+ and CD8+ T cells. Naïve cells were classified as CD45RA CCR7; effector memory cells as CD45RA CCR7; central memory cells as CD45RA CCR7; and effector cells as CD45RA CCR7.
(TIF)

S1 Table Supporting Table 1: Antibodies and clones used for *exvivo* analysis. This table shows the antibodies and clones used for the *exvivo* analysis.
(DOC)

S2 Table Supporting Table 2: Definition for memory T cell subsets. This table indicates the definitions for memory T cell subsets of naïve cells, Central memory cells, Effector memory cells, Effector cells based on the expression of CD45RA and CCR7.
(DOC)
