# Supplementary figures and images for: Altered levels of memory T cell subsets and common γc cytokines in Strongyloides stercoralis infection and partial reversal following anthelmintic treatment
Source: PLoS Negl Trop Dis. 2018 May 24;12(5):e0006481. doi: 10.1371/journal.pntd.0006481 (PMC5991401; doi:10.1371/journal.pntd.0006481)

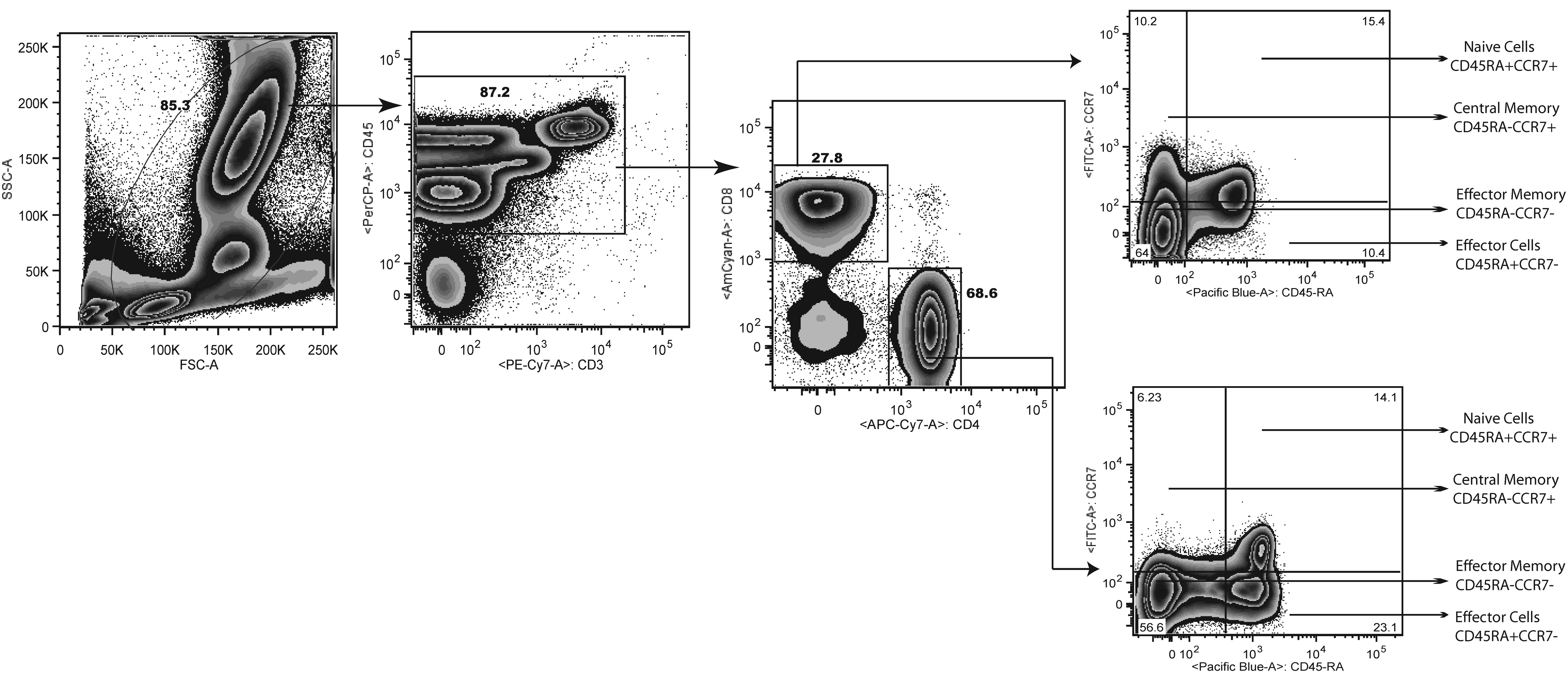

Supplement: S1 Fig — Naïve cells were classified as CD45RA CCR7; effector memory cells as CD45RA CCR7; central memory cells as CD45RA CCR7; and effector cells as CD45RA CCR7. (TIF) [file pntd.0006481.s001.tif]
