# Supplementary material for: Altered levels of memory T cell subsets and common γc cytokines in Strongyloides stercoralis infection and partial reversal following anthelmintic treatment
Source: PLoS Negl Trop Dis. 2018 May 24;12(5):e0006481. doi: 10.1371/journal.pntd.0006481 (PMC5991401; doi:10.1371/journal.pntd.0006481)
Supplement: S1 Table — This table shows the antibodies and clones used for the exvivo analysis. (DOC) [file pntd.0006481.s002.doc]

**Supplementary Table I: Antibodies and clones used for Exvivo analysis**

| Antibody | Flurochrome | Clone | Company |
| --- | --- | --- | --- |
| CD3 | AmCyan | clone SK7 | BD |
| CD4 | phycoerythrin (PE) Cy7 | SK3 | BD |
| CD8 | allophycocyanin (APC) H7 | SK1 | BD |
| CD45RA | Pacific Blue | H1100 | Biolegend |
| CCR7 | FITC | 3D12 | eBioscience |
