# Supplementary material for: Altered levels of memory T cell subsets and common γc cytokines in Strongyloides stercoralis infection and partial reversal following anthelmintic treatment
Source: PLoS Negl Trop Dis. 2018 May 24;12(5):e0006481. doi: 10.1371/journal.pntd.0006481 (PMC5991401; doi:10.1371/journal.pntd.0006481)
Supplement: S2 Table — This table indicates the definitions for memory T cell subsets of naïve cells, Central memory cells, Effector memory cells, Effector cells based on the expression of CD45RA and CCR7. (DOC) [file pntd.0006481.s003.doc]

**Supplementary Table II: Definition for Memory T cell subsets**

| **Subsets** | **Parameter** |
| --- | --- |
| CD45RA+ CCR7+ | Naïve cells |
| CD45RA-CCR7+ | Central memory |
| CD45RA-CCR7- | Effector memory |
| CD45RA+CCR7- | Effector cells |
